# Prediction of Cognitive Decline in Temporal Lobe Epilepsy and Mild Cognitive Impairment by EEG, MRI, and Neuropsychology

**DOI:** 10.1155/2020/8915961

**Published:** 2020-05-20

**Authors:** Yvonne Höller, Kevin H. G. Butz, Aljoscha C. Thomschewski, Elisabeth V. Schmid, Christoph D. Hofer, Andreas Uhl, Arne C. Bathke, Wolfgang Staffen, Raffaele Nardone, Fabian Schwimmbeck, Markus Leitinger, Giorgi Kuchukhidze, Marlene Derner, Jürgen Fell, Eugen Trinka

**Affiliations:** ^1^Faculty of Psychology, University of Akureyri, Akureyri, Iceland; ^2^Department of Neurology, Centre for Cognitive Neuroscience, Paracelsus Medical University, Salzburg, Austria; ^3^Spinal Cord Injury and Tissue Regeneration Center, Paracelsus Medical University, Salzburg, Austria; ^4^Multimedia Signal Processing and Security Lab, Department of Computer Sciences, Paris Lodron University, Salzburg, Austria; ^5^Research Group Statistics and Probability, Department of Mathematics, Paris Lodron University, Salzburg, Austria; ^6^Department of Neurology, F. Tappeiner Hospital, Merano, Italy; ^7^Department of Epileptology, University of Bonn, Bonn, Germany

## Abstract

Cognitive decline is a severe concern of patients with mild cognitive impairment. Also, in patients with temporal lobe epilepsy, memory problems are a frequently encountered problem with potential progression. On the background of a unifying hypothesis for cognitive decline, we merged knowledge from dementia and epilepsy research in order to identify biomarkers with a high predictive value for cognitive decline across and beyond these groups that can be fed into intelligent systems. We prospectively assessed patients with temporal lobe epilepsy (*N* = 9), mild cognitive impairment (*N* = 19), and subjective cognitive complaints (*N* = 4) and healthy controls (*N* = 18). All had structural cerebral MRI, EEG at rest and during declarative verbal memory performance, and a neuropsychological assessment which was repeated after 18 months. Cognitive decline was defined as significant change on neuropsychological subscales. We extracted volumetric and shape features from MRI and brain network measures from EEG and fed these features alongside a baseline testing in neuropsychology into a machine learning framework with feature subset selection and 5-fold cross validation. Out of 50 patients, 27 had a decline over time in executive functions, 23 in visual-verbal memory, 23 in divided attention, and 7 patients had an increase in depression scores. The best sensitivity/specificity for decline was 72%/82% for executive functions based on a feature combination from MRI volumetry and EEG partial coherence during recall of memories; 95%/74% for visual-verbal memory by combination of MRI-wavelet features and neuropsychology; 84%/76% for divided attention by combination of MRI-wavelet features and neuropsychology; and 81%/90% for increase of depression by combination of EEG partial directed coherence factor at rest and neuropsychology. Combining information from EEG, MRI, and neuropsychology in order to predict neuropsychological changes in a heterogeneous population could create a more general model of cognitive performance decline.

## 1. Introduction

Epilepsies and dementia are contributing substantially to the world's global burden of disease [1]. In 2005, there was an estimate of more than 50 million people living with active epilepsy [2]. In 2015, over 46 million people lived with dementia, and this number is estimated to increase to 131.5 million by 2050 [3]. The risk of unprovoked seizures in Alzheimer's dementia is eight to tenfold higher than in the general population [4–6]. Alzheimer's disease, mild cognitive impairment, and temporal lobe epilepsy share not only core symptoms, such as cognitive dysfunction, but also hippocampal atrophy [7]. Conversion rates from mild cognitive impairment to Alzheimer's disease within 30 months are 48.7% for amnestic subtype and 36.8% for nonamnestic subtype [8]. By contrast, the question whether patients with temporal lobe epilepsy can suffer from a progressive cognitive decline or whether most of the cognitive deficits are acquired at the initial insult, which cause both temporal lobe epilepsy and cognitive deficits, is far from clear [9–12]. The potential contributors to cognitive decline in temporal lobe epilepsy could be seizures, interictal epileptiform events, or other mechanisms [13].

Studies on seizures in early Alzheimer's disease suggest that they may be the harbinger of cognitive decline [14, 15]. The lifetime prevalence of seizures in mild cognitive impairment/Alzheimer's disease varies considerably between studies [16, 17]. Routine electroencephalography (EEG) detects interictal epileptiform activity in more than 40% of patients with Alzheimer's disease [18]. Precise assessment is challenging because nonconvulsive seizures are hard to recognize, especially in confused patients [19–21]. Cognitive decline occurs earlier in patients with mild cognitive impairment/Alzheimer's disease when patients encounter seizures, especially, when there is subclinical epileptiform activity in the temporal lobe [14, 15, 18].

In humans, functional magnetic resonance imaging (MRI) studies disclosed hippocampal hyperexcitability, which was reversed under antiepileptic treatment [21, 22]. More recently, Lam et al. [23] detected scalp-silent mesial temporal epileptic activity using intracranial electrodes, providing evidence for the contribution of hippocampal excitability in the prodromal stage of Alzheimer's disease. These findings suggest that there might be considerable overlap in the pathological mechanisms underlying cognitive decline in both diseases: temporal lobe epilepsy and mild cognitive impairment/Alzheimer's disease, and it is a valid question whether there exist generic biomarkers as a common denominator for progression of cognitive decline.

Brain volumetry using MRI has been used to determine whether the condition is progressive or not in mild cognitive impairment/Alzheimer's disease and in temporal lobe epilepsy [24], but these studies never searched for similarities in cognitive decline between the two disorders [7]. Based on the large database of Alzheimer's Disease Neuroimaging Initiative, conversion from mild cognitive impairment to Alzheimer's disease was predictable with an accuracy of 0.7–0.79 [25]. Structural measures in the temporal lobe and its subregions such as the hippocampus or the entorhinal cortex seem to be highly indicative for progression of the disease [26–32]. It was early suggested that automation of volumetric assessment could pave the way for clinical implementation of prognostic consulting in the clinical setting [33]. Moreover, wavelet features from the MRI have been applied for classifying patients with Alzheimer's dementia and mild cognitive impairment [34–36].

Despite the considerable attention attracted by neuroimaging in the past decade [37], clinical use of the EEG for diagnosis and prognosis of dementia was emphasized more than 30 years ago [38]. After seminal work on the predictive value of qualitative EEG analysis for the progression of memory decline in Alzheimer's disease [39, 40], it was suggested to use quantitative EEG for the prediction of cognitive decline [41, 42]. EEG properties correlate with genetic biomarkers [43] and biological markers of microvascular degeneration [44]. Event-related EEG potentials [45–52] or bandpower in the delta, theta, alpha, and beta range [49, 53–61] have been utilized to predict cognitive decline. It seems that the EEG is most useful for predicting cognitive decline when being recorded during cognitive activation, allowing to measure the brain's response to cognitive effort [62]. Analyses of brain networks from the EEG were also implemented in prognostic studies [63] with a distinction between stable mild cognitive impairment and progression to Alzheimer's disease by up to 86% [64] and in differentiating normal elderly people with subjective cognitive complaints with vs. without progression of cognitive symptoms by 90% [65].

Besides advanced analysis of brain activity, behavioral alterations predict future decline of cognitive functions. That is, impairment in cognitive domains as detected by neuropsychological assessment is indicative for further deterioration [66–72]. Moreover, depressive symptoms were found to be linked to the progression from mild cognitive impairment to Alzheimer's disease [73, 74]. It is therefore beneficial to include cognitive assessment and depression scores in prognostic studies.

Finally, multimodal assessment is being suggested to increase the accuracy of prognosis because different assessment modalities such as structural MRI and functional EEG may complement each other [75]. It was found that simple power analysis of the EEG in the sense of bandpower ratios was superior to hippocampal volume and led to a prognostic accuracy of 88.3% for conversion to Alzheimer's disease [76].

In the presented prospective study, we selected candidate biomarkers from both the resting and cognitive EEG, alone or in combination with MRI volumetric features and/or neuropsychological scores at baseline, in order to predict decline on neuropsychological subscales in patients with mild cognitive impairment, temporal lobe epilepsy, and subjective cognitive complaints and healthy controls. By fitting one model to these populations, we would like to identify predictive biomarkers that are not restricted to one neurological population but which could be generic markers of cognitive decline.

## 2. Methods

### 2.1. Ethics Approval and Consent to Participate

The study was approved by the local Ethics Committee (Ethics Commission Salzburg/Ethikkommission Land Salzburg; number 415-E/1429) and was designed according to the Declaration of Helsinki. Written informed consent was obtained from all participants. Healthy participants were remunerated for their expenditure of time.

### 2.2. Sample and Recruitment

Patients with amnestic mild cognitive impairment and amnestic subjective cognitive complaints were recruited in the memory outpatient clinic of the Department of Neurology, Christian Doppler Medical Centre, Paracelsus Medical University Salzburg, Austria. The diagnosis was assigned by the medical doctor according to the results of the described multimodal examination according to the criteria of Petersen [77]. We conformed to the definition where amnestic mild cognitive impairment equals to level three and amnestic subjective cognitive complaints equals to level two of the global deterioration scale for aging and dementia [78–80]. The diagnosis of amnestic mild cognitive impairment and amnestic subjective cognitive complaints indicates that the complaints and/or deficits were detectable only in the memory domain and not on other cognitive subscales.

The multimodal neurological assessment included imaging (3T magnetic resonance tomography, and in suspicious cases also single photon emission computed tomography with T c99-hexamethylpropylenaminooxim, for cerebral blood perfusion) and neuropsychological testing. We excluded patients when inflammatory, vascular, metabolic, or traumatic causes, as well as major depression, psychosis, or any pharmacological therapy, could better explain cognitive impairment or cognitive complaints.

Patients with refractory unilateral temporal lobe epilepsy were recruited in the epilepsy outpatient clinic of the Department of Neurology, Paracelsus Medical University Salzburg, Austria. Diagnosis was based on multimodal neurological assessment, including imaging (3T magnetic resonance tomography and single photon emission computed tomography with Tc99-hexamethylpropylenaminooxim), neuropsychological testing, and video EEG examination for up to five days. We excluded patients with progressive lesions or immunological causes of epilepsy.

The sample of healthy participants was recruited among the students of the Paris Lodron University of Salzburg, Austria, as well as among senior citizens associations in order to closely resemble the age range of the patient groups. Healthy participants were free of a history for neurological or psychiatric diseases and were not receiving any psychoactive medication.

### 2.3. MRI

T1-weighted MRI volumes were acquired using a Siemens (Erlangen, Germany) Magnetom TrioTim syngo MR B17 at 3 Tesla, a 12-channel head coil, and the following parameters: sagittal orientation, 192 slices per slab, 256 mm FoV read at 93.8%phase, TR (repetition time) = 2300 ms, TE (echo time) = 2.91 ms, TI (inversion time) = 900 ms, FA (flip angle) = 9 deg, and a slice thickness of 1 mm resulting in a voxel size of 1 × 1 × 1 mm^3^.

We extracted three types of features from MRI data: volumetry, local binary patterns, and wavelets.

#### 2.3.1. MRI Volumetry

The automated segmentation was performed using a set of 30 hand labeled atlases (83 regions each, for a list of the regions, see Supplementary material) made publicly available by Hammers et al. [81]. After brain extraction using the brain extraction tool in the functional MRI of the Brain Software Library (available from http://fsl.fmrib.ox.ac.uk), all participants were diffeomorphically registered using advanced normalization tools (available from http://stnava.github.io/ANTs/) to each atlas. The final segmentation was obtained by using majority voting to fuse the registration outcomes for each subject. The result was a labeled volume, containing labels for the 83 cortical and subcortical structures.

The volume of the segmented regions was normalized by the sum of all volumes, that is, we calculated the percent each region took in relation to the sum of all segmented regions. This comes down to scaling to global brain volume, which is different to the more usual approach of scaling to total intracranial volume (including the CSF space) as a measure of head size. Total intracranial volume is usually not affected by disease, whereas global brain volume decreases with disease severity in neurodegenerative conditions.

#### 2.3.2. Local Binary Patterns

Local binary patterns can be used to describe two-dimensional textures. According to [82], we calculated a local binary pattern descriptor for the three-dimensional MRI data. In short, for each voxel, the three planes spanned by the coordinate system are used to extract two-dimensional local binary patterns. The three resulting histograms are then concatenated to a single feature vector. In our setup, we slightly modified this approach in order to get a region-based local binary pattern feature representation. For this purpose, the local binary pattern volume is masked to the specific region before histogram calculation and concatenation. The final output is a feature vector per subject per region.

### 2.4. Wavelets

The discrete wavelet transform has a long history in two-dimensional image representation and texture analysis [83]. It takes advantage of decomposing the image into a detail (high-pass) and an approximation (low-pass) part on multiple resolutions. In our setup, we used the stationary discrete wavelet transform [84] to extract feature representations of different resolution and detail level from each three-dimensional volume.

The feature volumes are then masked to the individual brain regions and the wavelet coefficients in the masked region represented by an estimated generalized Gaussian [85]. The concatenation of the estimated parameters (of the generalized Gaussian) over all resolutions and detail/approximation combinations leads to the final feature vector.

### 2.5. Neuropsychological Assessment

Neuropsychological assessment was performed at baseline and at 18-month follow-up. The test battery included matrices, mosaics, and repeating numbers from Wechsler's intelligence test [86], a verbal memory test [87], the diagnosticum for cerebral damage [88], the Regensburg verbal fluency test [89], the test for attentional performance flexibility [90], and Beck's Depression Inventory [91]. In addition, healthy controls were examined with the Montreal cognitive assessment [92], in order to screen for unknown cognitive impairment.

From the neuropsychological test results, we formed domain-specific composite scores which indicated whether a participant had a decline of at least one standard deviation according to the test manuals on one of the included subscales. Subjects without a decline included those who did not show a significant decline as well as those who showed improvement. We included only domains for the purpose of the prediction analysis in which the number of participants with a decline was large enough for the machine learning and cross validation algorithm. For the purpose of classification analysis, the number of participants must be equally balanced in the two groups that should be classified, that is, in this case, the participants with vs. without decline. Therefore, we included composite scores of decline based on z-scores in the following cognitive domains:

- Executive functions, based on 7 scales: matrices and repeating numbers from 9.
- Wechsler's intelligence test [86], Regensburg verbal fluency test version 1 and 2, subscales formal lexical verbal fluency, semantic categorical fluency, and semantic category transition [89], test for attentional performance, and subscale summary score for flexibility [90].
- Visual and verbal memory, based on 5 scales, of which 4 are from the verbal memory test [87], that is, learning, consolidation, recall, and recognition, and the fourth is the summary score of the diagnosticum for cerebral damage [88].
- Divided attention based on the two summary scales from the test for attentional performance flexibility [90] and summary score of two tasks for divided attention.

Moreover, we included depression as a factor that could be predicted, in order to extend previous research that predicted decline based on depressive symptoms at baseline.

### 2.6. EEG Procedure and Task

Recordings started with a resting condition which lasted for 2-3 min. After that, the first session included the learning condition of 72 pairs of German nouns, immediately followed by the cued recall and recognition. A second EEG session took place around 2 weeks later and consisted only of rest, cued recall, and recognition. Each task was preceded by a training session that included written instruction on the screen. Each trial ended with the participant's response, followed by an intertrial interval of 1 s. Participants were seated in front of a desktop in order to allow a familiar distance to the screen and comfortable reach to the keyboard. All participants had normal or corrected to normal vision (glasses or contact lenses).

The task was presented on a standard 19″ screen and prepared in Presentation (Neurobehavioral Systems Inc.). The stimuli were centered text in black letters on a white screen in font Arial, 48 pt.

### 2.7. EEG Registration

EEG was recorded in a quiet room using a BrainCap with a 10–20 system and a BrainAmp (Brain Products GmbH, Germany) 16-bit analog-to-digital converting amplifier. The sampling rate was 500 Hz. Of the 32 recorded channels, one was used to monitor the lower vertical electrooculogram and one was used to measure electrocardiographic activity. Two channels were positioned at the earlobes for rereferencing purposes to remove the bias of the original reference, which was placed at FCz. Data analysis was conducted for a subset of 17 electrodes: F3, F4, C3, C4, P3, P4, O1, O2, F7, F8, T7, T8, P7, P8, Fz, Cz, and Pz. Impedances were kept below 10 kΩ.

The two EEG sessions were arranged to take place at the same time of the day. For most participants, EEG was performed within the same time range around noon (1 pm).

### 2.8. Data Preparation

Data were preprocessed with Brain Vision Analyzer (Version 1.05.0005, Brain Products GmbH). In order to rereference all channels, a new reference was built by averaging the signal of earlobe electrodes. Butterworth zero-phase filters were used for a high-pass filter from 1 Hz (time constant 0.1592 s, 48 dB/oct), and an additional notch filter (50 Hz) was applied.

An automatic artefact detection was carried out in order to exclude artefacts. Maximal allowed voltage step per sampling point was 50 *μ*V (values which exceeded this threshold were excluded within a range of ±100 ms), maximal allowed absolute difference on an interval of 200 ms was 200 *μ*V, and lowest allowed absolute difference during an interval of 100 ms was 0.5 *μ*V (values which exceeded this were marked with a surrounding of ±500 ms).

The preprocessed data were exported into a generic data format and imported to Matlab (release R2017b, Mathworks, Massachusetts, USA).

The data were segmented into 1000 ms segments (i.e., 500 sampling points) for each participant and each condition. For each condition (learning/recall), the segment started 100 ms before stimulus onset and ended 900 ms afterwards. If the segment overlapped with a marked artefact, it was excluded.

### 2.9. Measures of Interaction

For each segment, we estimated a set of measures of interaction between the selected 17 channels. The measures were calculated with the functions mvfreqz.m and mvar.m from the BioSig toolbox [93] with a model order of 10. To estimate the multivariate autoregressive model, we used partial correlation estimation with unbiased covariance estimates [94]. The multivariate parameters in the frequency domain that can be derived from these transfer functions were computed for 1 Hz frequency steps between 2 and 125 Hz. The following measures were extracted: auto and cross spectrum [95], direct causality [96], transfer function [97], transfer function polynomial [97], real- and complex-valued coherence [98], partial coherence [99], partial directed coherence and partial directed coherence factor [100], generalized partial directed coherence [101], directed transfer function [102], direct directed transfer function and full frequency directed transfer function [103], and Geweke–Granger causality [104, 105].

We averaged the measures in classical frequency ranges delta (2–4 Hz), theta (5–7 Hz), alpha (8–13 Hz), beta (14–30 Hz), gamma (31–80 Hz), and high gamma (81–125 Hz). We concatenated all of the nonredundant values from these interaction matrices for all frequencies of interest.

### 2.10. Statistical Analysis

Age was compared between groups with a classical univariate ANOVA. Equality of distribution of women and right-handed participants across groups was assessed with a chi-squared test.

The neuropsychological characteristics of the subgroups at inclusion time and at follow-up were evaluated using a nonparametric repeated measures ANOVA with a parametric bootstrap [106] with factor group and the two neuropsychological assessments (baseline, 18-month follow-up) as within subject factor. We chose a semiparametric repeated measures MANOVA that only requires metric data, but allows for nonnormality and variance heterogeneity [106]. This method is implemented in the R-package MANOVA.RM [107]. We used it with the parametric bootstrap which showed the most favourable performance in unbalanced designs and was therefore generally recommended [106].

### 2.11. Classification Analysis

We conducted classification of participants with vs. without decline on the respective subscales with all possible combinations of feature vectors. A detailed list is included in the Supplementary Section.

For the EEG, we could augment the sample size by considering each segment individually for the classification. That is, upon classification, we created one sample for each EEG segment. This way, we obtained, e.g., a maximum of 72 samples per participant for the learning, recall, and recognition conditions. For the rest data, the maximum number was 180 samples for the 3 min of rest. Note that depending on the segments that were excluded because of artefacts, these numbers could be significantly lower. We assumed that this would increase the robustness of the model, since we considered the intraindividual variance of the EEG. When these values were combined with MRI or neuropsychology, we simply added the same MRI feature vector or neuropsychological feature vector to each of the EEG feature vectors. This was done because an accurate noise model that would allow for classical data augmentation could not be estimated reliably based on the small dataset. This is a rather conservative approach and at worst would lead to low classification accuracies.

For the k-fold cross validation, we grouped the segments participantwise, so that all segments of one patient were included in one partition. This rather conservative approach was chosen because it can be assumed that the features are more similar within one participant than between participants.

We performed a classification in the sense of supervised learning with a linear kernel function (dot product) and quadratic programming in order to find the separating hyperplane, resulting in a 2-norm soft-margin support vector machine, by using the Matlab functions svmtrain and svmclassify from the statistics and machine learning toolbox.

### 2.12. Cross Validation and Feature Subset Selection

It is known that when this length exceeds the size of the sample, it can cause artificially high accuracies due to overfitting. Because of the high dimensionality of the data, we implemented a feature subset selection procedure in order to limit the feature vectors to a maximum length of 30, i.e., approximately two-thirds of the size of the sample.

Classification and feature subset selection was done in a nested design with 3 layers with 5-fold cross validation (an illustration can be found in Figure 1 in the Supplementary section). We implemented an outer layer as a division of the data into 20% of the data for testing the resulting model and 80% for feature vector optimisation and cross validation, i.e., submitted to the middle layer. The middle layer is a first inner loop, implemented again with 5-fold cross validation. This loop aims to estimate the consistency of selected features, since each run yields a different feature vector. The inner layer is a second, thus, nested inner loop, again with 5-fold cross validation in order to perform adequate feature subset selection. So-called k-fold cross validation consists of *k* repetitions of leaving out *N*/*k* samples as the training set, while the remaining *N* − (*N*/*k*) samples are used during the training step.

More details about the algorithm are described in the Supplementary materials.

## 3. Results

### 3.1. Sample

We recruited a total sample of 71 participants from May 2012 to December 2015. Out of these, there were 20 patients with mild cognitive impairment, seven patients with subjective cognitive complaints, 17 patients with temporal lobe epilepsy (eight right-lateralized temporal lobe epilepsy; nine left-lateralized temporal lobe epilepsy), and 26 healthy controls. Follow-up of the neuropsychological examination after 18 months was obtained from 51 patients. The largest proportion of dropouts occurred in the temporal lobe epilepsy group, where several patients underwent surgical intervention and were therefore excluded from further analysis because the resection of brain tissue would interfere with the spontaneous or disease-associated development of cognitive performance.

The patients' characteristics are given in Table 1. The groups did not differ significantly in sex (*χ*^2^(4) = 7.04; *p*=0.15) and right-handedness (*χ*^2^(4) = 3.67; *p*=0.45), but different in terms of age (*F*(4, 46) = 10.43; *p* < 0.001). This was mainly due to the very young group of patients with right-lateralized temporal lobe epilepsy (coefficients *t* − value = −4.54; *p*=0.00004) and the older mild cognitive impairment group (coefficients *t* − value = 2.14; *p*=0.04).

Supplementary Tables S1–S4 provide further details on participants recorded at baseline.  Table S1 provides the results of assessment of pathological findings within the hippocampus based on structural MRI by a board certified neuroradiologist, Table S2 provides self-reported medication of all participants in this study, Table S3 provides the results of the assessment of pathological findings and signs of sleepiness in the EEG by a board certified neurophysiologist, and Table S4 provides clinical aspects of patients with epilepsy, specifically whether seizures occurred within 24 h before or after the EEG recording took place.

### 3.2. Cognitive Decline

Test results for the effects of group and cognitive changes over the 18-month follow-up, as well as interaction between group and change, are given in Table 2, and average raw scores on the scales per subgroup are given in Supplementary Table S5. Montreal cognitive assessment in healthy controls resulted in an average score of 27.78 (SD = 1.59).

On all subscales except Beck's Depression Inventory, healthy controls showed the highest values, except for the group of patients with subjective cognitive complaints, who performed even better on some subscales. Patients with temporal lobe epilepsy scored lowest on the intelligence test, verbal fluency, attentional performance, and on the percentile rank of the diagnosticum for cerebral damage. For verbal memory, the means were comparable for patients with temporal lobe epilepsy and mild cognitive impairment. Finally, patients with temporal lobe epilepsy showed the highest scores on Beck's Depression Inventory. In summary, patients with temporal lobe epilepsy were broadly affected on all assessed scales, while patients with mild cognitive impairment and subjective cognitive complaints were mainly affected in the verbal memory test.

After Bonferroni correction, the effects need to be interpreted at *p* < 0.002. Decline was significant for categorical fluency. None of the interactions of group and decline was significant. There were group effects in intelligence subscales, verbal fluency, verbal memory consolidation, visual reaction, depression, and in the percentile rank of the diagnosticum for cerebral damage.

By means of our definition of relevant decline, Table 3 indicates the numbers of patients or participants in each subgroup of the assessed sample. In absolute numbers, on all scales but for the worsening of depression, there were more patients with mild cognitive impairment with decline than without cognitive decline, while for all other groups, the number of patients or participants without decline was equal or larger than the number with decline.

### 3.3. Prediction of Cognitive Decline

We separately evaluated classification accuracy for participants with and without decline, which can be understood as sensitivity (how many participants with decline were classified correctly) and specificity (how many participants without decline were classified correctly). If one of these two values was below 70%, we concluded that the result was rather a statistical artefact of the classifier to be biased towards one of the two groups. We chose the rather strict benchmark of 70% because in some prediction scenarios, the sample sizes were misbalanced as can be seen in Table 3. The worst misbalancing was obtained for prediction of a significant change in depression, where approximately only 15% showed a worsening at follow-up. Since the other groups were almost equally balanced with, e.g., 46% of patients with a decline in visual-verbal memory and in order not to be too strict, we chose 70% as a compromise.

In Table 4, we list the results where sensitivity and specificity were at least 70%. Supplementary Table S6 provides numbers of samples for each of these results and the subgroups with and without decline, and Table S7 provides accuracy, sensitivity, and specificity for subgroups. Most of these effective predictions combine at least 2 modalities, thus suggesting the importance of multimodal assessment.

Structural MRI was used in combination with partial coherence from EEG during recognition or recall with or without neuropsychological baseline testing in order to predict executive function decline. The selection of neuropsychological measures is illustrated in Supplementary Figure 2. Most selected subscales except for Beck's Depression Inventory, recognition of verbal material, and the scales for divided attention included tests that were included in the executive function scale. Thus, executive function decline was predicted by the baseline in this cognitive domain, combined with measures of divided attention, intelligence, depression, and verbal memory.

**Figure 1:**
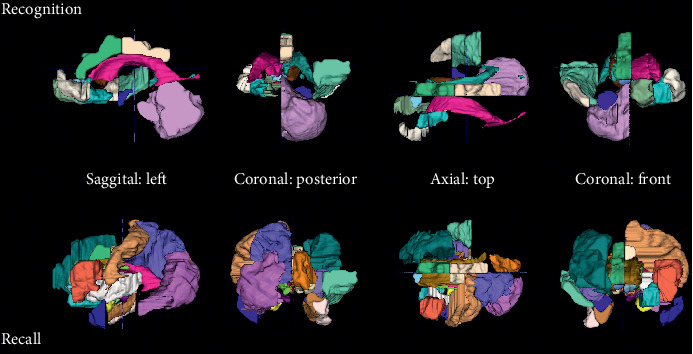
MRI regions selected for prediction of executive functions decline. For the combination with EEG during recognition and neuropsychology, volumetry from the following regions was selected as features: amygdala right, caudate nucleus right, medial, lateral, and posterior orbital gyrus left, superior temporal gyrus middle part right, bilateral lateral ventricle excluding temporal horn, subcallosal area left, bilateral pre-subgenual frontal cortex left, cerebellum right, superior temporal gyrus anterior part right, and anterior and posterior cingulate gyrus right. For the combination with EEG during recall without neuropsychology, volumetry from the following regions was selected as features: bilateral amygdala, anterior temporal lobe lateral part left, parahippocampal and ambient gyrus right, superior temporal gyrus middle part right, fusiform gyrus right, bilateral insula, lateral remainder occipital lobe left, anterior cingulate gyrus right, bilateral posterior cingulate gyrus, middle frontal gyrus right, bilateral caudate nucleus, bilateral nucleus accumbens, thalamus right, corpus callosum, lateral ventricle excluding temporal horn left, inferior frontal gyrus left, postcentral gyrus left, superior parietal gyrus left, cuneus right, posterior orbital gyrus left, substantia nigra left, subgenual frontal cortex left, subcallosal area right, and bilateral pre-subgenual frontal cortex L.

The MRI features that were selected for prediction of executive function decline in combination with EEG during recognition and neuropsychology or recall without neuropsychology are shown in Figure 1.

Without neuropsychology, more and also different regions from MRI were selected. For example, when focusing on the frontal region, while the combination with neuropsychology included only the bilateral pre-subgenual frontal cortex left, the classification without neuropsychology included the middle frontal gyrus right, the inferior frontal gyrus left, and the subgenual frontal cortex left.

**Figure 2:**
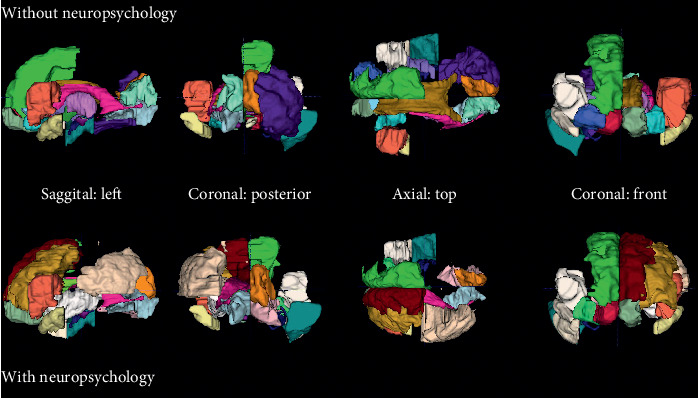
MRI regions selected for prediction of visual-verbal function decline. The following regions were selected for wavelet features: middle and inferior temporal gyrus right, insula right, lateral remainder occipital lobe right, thalamus left, corpus callosum, lateral ventricle excluding temporal horn left, lateral ventricle temporal horn right, third ventricle, anterior orbital gyrus right, bilateral inferior frontal gyrus, superior frontal gyrus right, lingual gyrus left, bilateral cuneus, bilateral medial orbital gyrus, posterior orbital gyrus left, bilateral substantia nigra, bilateral subgenual frontal cortex, bilateral subcallosal area, bilateral pre-subgenual frontal cortex, and bilateral superior temporal gyrus anterior part. For the combination with neuropsychology, the following regions were selected for wavelet features: anterior temporal lobe lateral part right, middle and inferior temporal gyrus right, insula left, middle frontal gyrus left, inferolateral remainder parietal lobe left, caudate nucleus left, lateral ventricle excluding temporal horn left, lateral ventricle temporal horn right, third ventricle, bilateral inferior and superior frontal gyrus, bilateral lingual gyrus, cuneus right, bilateral medial orbital gyrus, posterior orbital gyrus right, substantia nigra left, subgenual frontal cortex right, bilateral subcallosal area, bilateral pre-subgenual frontal cortex, and bilateral superior temporal gyrus anterior part.

For visual-verbal memory, classification accuracy based on wavelet features from the MRI was at 80% without and 86% with neuropsychological measures. Only two neuropsychological measures were selected (recall on the verbal learning and memory test and errors on the test for flexibility in attentional performance), and they were selected only in one out of 5 cross validation runs. The regions from which the MRI-wavelet features were extracted are shown in Figure 2.

The two selections overlapped to a large extent. However, occipital regions were included to a lesser extent when neuropsychological features were included and frontal regions were included to a larger extent alongside neuropsychological features. It needs to be considered that no patients with temporal lobe epilepsy contributed to this classification (see Supplementary Table 7).

**Figure 3:**
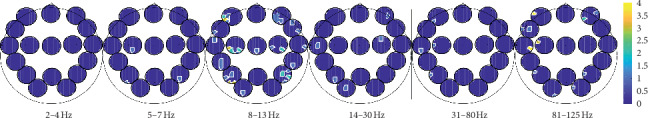
EEG features selected for prediction of divided attention decline. Imaginary coherence topoplots during rest in 6 frequency ranges. The colors indicate the selection of the respective areas/frequencies for prediction of executive function decline. That is, the color from dark blue (0) to yellow (4) indicates how often the respective regional interaction in a specific frequency was selected in the 5 cross validation runs (4 was the maximum). The higher the number of selections, the more reliable the result. The topographical representation illustrates the interaction matrix of all possible combinations of electrodes. The head is represented as the large circle and the electrode positions as smaller circles on this large circle. The colored small circles indicate again a head, so that connections between the respective region on the large circle and the colored region in the small circle can be inferred.

Divided attention decline was predicted with wavelet features and one neuropsychological feature (errors on flexibility in the test for attentional performance flexibility) by 81% and by structural MRI analysis, i.e., volumetry, and EEG imaginary coherence by 79%.  Figure 3 shows that the interaction of brain regions in the alpha frequency range is most indicative, i.e., most features were selected from this frequency range.

**Figure 4:**
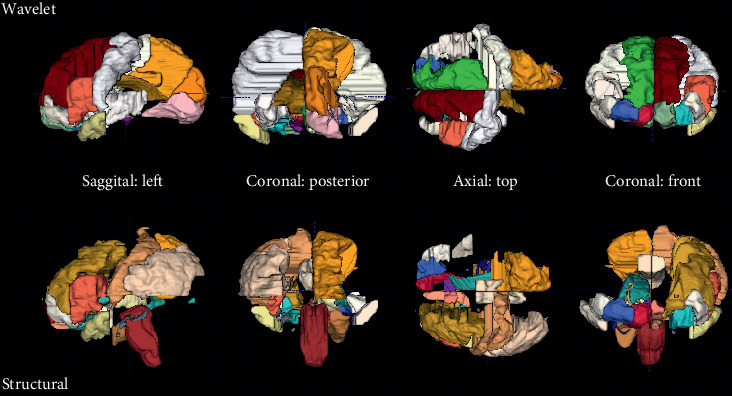
MRI regions selected for prediction of divided attention decline. For the prediction of divided attention decline, wavelet MRI features were selected from the following regions: insula left, corpus callosum, third ventricle, precentral gyrus left, straight gyrus right, anterior orbital gyrus right, bilateral inferior frontal gyrus, bilateral superior frontal gyrus, postcentral gyrus right, superior parietal gyrus right, lingual gyrus right, cuneus right, bilateral medial orbital gyrus, bilateral lateral orbital gyrus, posterior orbital gyrus left, bilateral substantia nigra, bilateral subgenual frontal cortex, bilateral subcallosal area, bilateral pre-subgenual frontal cortex, and bilateral superior temporal gyrus anterior part. For the prediction of divided attention decline by structural MRI volumetry, the following regions were selected: parahippocampal and ambient gyrus right, brainstem excluding substantia nigra, bilateral posterior cingulate gyrus, middle frontal gyrus left, inferolateral remainder parietal lobe left, nucleus accumbens right, thalamus right, lateral ventricle including temporal horn right, lateral ventricle temporal horn left, straight gyrus left, anterior orbital gyrus right, inferior frontal gyrus left, postcentral gyrus left, superior parietal gyrus right, medial orbital gyrus right, bilateral lateral orbital gyrus, posterior orbital gyrus left, bilateral subgenual frontal cortex, subcallosal area right, and bilateral superior temporal gyrus anterior part.

The interactions selected within this range represent frontal to parietooccipital, central to parietooccipital, within posterior, and within occipital interdependencies. The MRI regions selected for prediction of divided attention decline are represented in Figure 4.

The wavelet features were extracted from classical regions known to be important for divided attention, such as the bilateral inferior and superior frontal gyri and the bilateral pre-subgenual and subgenual frontal cortex. In contrast, in combination with the EEG, only the volumes of the inferior frontal gyrus left and the bilateral subgenual frontal cortex were selected.

Depression was predicted at 83% by partial directed coherence factor at rest and neuropsychological scales. The subset of selected features from the partial directed coherence factor extended over a broad frequency range from theta to high gamma and was most pronounced for interactions with frontal left and parietal right signals. However, the maximum of cross runs in which such features were chosen was 2 out of 5, thus questioning the generalizability of these results. In contrast, the selection of neuropsychological subscales for the prediction of increase of depressive symptoms was more consistent, with two subscales reaching 5 out of 5 cross validation runs, and 3 others 4 out of 5 cross validation runs. The choice of neuropsychological subscales suggests that the predictability of depression is rather nonspecifically related to performance in various cognitive domains, such as executive functions, visual and verbal memory, intelligence, and attention. The Beck Depression Inventory score itself was also among the selected measures, but was chosen in 3 out of 5 cross validation runs only. Since the prediction of depression was rather a secondary outcome in this study, the selected features are represented in Supplementary Figure 3.

## 4. Discussion

In this research project, we elaborated general models for the prediction of decline in cognitive domains. We identified shared predictors in patients with prodromal dementia and with temporal lobe epilepsy. The prediction of decline after 18 months in cognitive domains of executive functions, visual-verbal memory, and divided attention involved characteristics from MRI. EEG characteristics were useful in prediction of executive function decline and depression, but not for visual-verbal memory, and to a minor extent for divided attention. Finally, neuropsychological baseline diagnostics were most informative for all domains but executive functions, for which a similar or even better prediction was also obtained without neuropsychological features. Moreover, we could show that not only depression at baseline predicts cognitive decline, but also depression is predictable by cognitive impairment at baseline and by EEG biomarkers.

These results suggest (1) that there exist some general characteristics that are shared among different neurological populations and (2) that multimodal assessment of these characteristics is useful for the prediction of neuropsychological changes.

### 4.1. Multimodal Assessment

Prediction of conversion from mild cognitive impairment to Alzheimer's disease was made based on neuropsychology with an accuracy of 76% [70] and based on MRI by 75.05% [108]. Given that cognitive decline can also be predicted by neuropsychological assessments at baseline [66, 67, 69, 71, 72] and that cognitive assessment is much cheaper than imaging, it seems justified to ask why the effort should be made to apply additional examinations of EEG or MRI.

Multimodal assessment has been demonstrated to be a valid approach for the prediction of disease progression in patients with mild cognitive impairment [109–113]. It was found that the combination of EEG with neuropsychological assessment increases the prognostic accuracy for cognitive decline [114], for example, at 78.5% [109]. Our results are well in line with these findings, with accuracies above those reported for prediction based on one modality. Moreover, we could also replicate the importance of depression for prediction of further decline [73, 74] and extend these findings by prediction of worsening of depressive symptoms. As for cognitive decline, worsening of depression is predicted by depressive symptoms at baseline, but also by dysfunctions in other cognitive domains. This is very plausible since the irreversible decay of one's own cognitive functioning is a change which is difficult to accept.

The EEG was discussed to be an important source of information for normal and pathological ageing such as mild cognitive impairment and Alzheimer's disease [63]. It was claimed that the contribution of the EEG to clinical appraisal of mild cognitive impairment or Alzheimer's disease and its progression should not be limited to the identification of comorbidity with epilepsy [115]. The predictive value of EEG biomarkers [62] or functional MRI biomarkers [32, 116–118] can be increased when they are acquired during performance of specific tasks. Our results do not perfectly line up with these findings. For executive function, the EEG measures obtained during recall or recognition were most helpful, but for divided attention or depression, rest was more valid. Well in line with our results, measures for executive functions predict cognitive decline [68].

For example, the use of dual-tree complex wavelet transforms resulted in a classification accuracy of Alzheimer's disease vs. healthy controls of approximately 93% in Alzheimer's Disease Neuroimaging Initiative dataset and 97% in the Open Access Series of Imaging Studies dataset [34]. However, classification into stages rather than into progression of decline is much easier to accomplish.

In general, by comparing our results to previous research, it must be considered that the reported accuracies of 70–79% based on Alzheimer's Disease Neuroimaging Initiative [25] were obtained for the clinical question of conversion from mild cognitive impairment to Alzheimer's disease, while the presented analysis aimed to predict progression in specific cognitive domains.

As an outlook, we may anticipate that not only other modalities but also other characteristics may be assessed by MRI or EEG. For instance, the assessment of structural connectivity by means of diffusion tensor imaging has proven to be especially useful to predict memory decline in patients at risk for Alzheimer's disease [119, 120].

### 4.2. Generalizability

An association between electroencephalographic markers and memory is far more established in epilepsy, than in mild cognitive impairment/Alzheimer's disease. For example, resting-state hippocampal theta connectivity obtained by magnetoencephalographic source reconstruction was found to be correlated with memory performance [121]. Moreover, there is a link between grey matter volume of the hippocampus, connectivity as revealed by fMRI, and memory performance [122] and even between hippocampal volume and structural connectivity [123]. A connectivity study based on the EEG identified the temporal lobe as an important node of brain networks involved in episodic memory retrieval [124]. The alterations in functional connectivity may be related to structural vulnerability due to epilepsy-associated damage, which can result in abnormal increase of neuronal connectivity [125]. Nevertheless, there are still many open questions with respect to a potential dementing course in subtypes of epilepsy and there exist no studies using these biomarkers in order to predict decline on memory subscales, except for studies assessing the mainly short-term effect of seizures and interictal epileptiform events [10, 13].

Machine learning is commonly used to predict progression of dementia [37, 109]. Nevertheless, the use of machine learning techniques needs to be done carefully, since many of the early studies using machine learning in neuroscience have not fully addressed the problem of overfitting the model to the training data, either because of the use of lengthy feature vectors that exceeded the size of the sample or because no strict separation between training and testing datasets was done. A special case of overestimation of the classification performance that is not easily recognized by the reader is when the features are preselected and then the preselected features are used for cross validation. In that case, the resulting accuracy may be much higher than that without the preselection, but the result is valid only for the present sample. Such a procedure sounds plausible for multimodal prediction [126], but leads to poor generalizability. For example, with our data sample, we ended up with accuracies close to 100 when we performed the feature selection separately for neuropsychology, MRI, and EEG, and then merged the resulting feature vectors. In contrast, the presented approach is much more strict and robust against overfitting.

### 4.3. Limitations

In our study, the sample of patients was rather small, especially for specific subsamples, such as patients with subjective cognitive complaints and patients with temporal lobe epilepsy. As an additional source of bias, the subgroups are unbalanced in terms of age, since naturally, mild cognitive impairment occurs in the elderly, while temporal lobe epilepsy may occur at all ages. This potential bias could be overcome in future studies with an older sample of temporal lobe epilepsy patients.

The first and main limitation is that only for the prediction of executive function decline, decline in divided attention, and worsening of depression, the sample size was large enough in all subgroups. The prediction of visual-verbal memory relied only on the groups of patients with mild cognitive impairment, subjective cognitive complaints, and healthy controls. The group of patients with temporal lobe epilepsy was more difficult to recruit, as these refractory patients often undergo surgical treatment, which meant that they were not eligible for the study at follow-up. Future studies should aim at multicenter recruitment to overcome this problem.

We found some variability in predictability of cognitive domain functioning depending on whether EEG data from the first or the second session were used. This might be an indicator of poor reliability or alternatively point towards some inherent properties of repeated testing of such a kind. For instance, we strongly assume that the first cued recall and recognition induced reconsolidation [127], thus affecting memory performance at the delayed session. This means that the second session's EEG pattern might be quite different from the first session and thus explains why the one or the other session is more informative.

Also relating to the two repeated sessions, we need to consider what happens in the period of 2 weeks. With respect to the temporal lobe epilepsy patients, we reported only seizure occurrence in proximity to the learning session. Patients with temporal lobe epilepsy may have experienced seizures during the 2 weeks, which may additionally have affected the recall. A valid documentation of the effect of seizures would require accurate EEG monitoring of ictal and interictal activity in the retention period. This is beyond the scope of the present work, but should be considered in future studies. The potential of interference between seizures and memory contents is still subject to debate [7].

Another factor that was not feasible to be recorded in a reliable way was a full record of medication in the follow-up period of 18 months. Alterations in medication from baseline could contribute to cognitive changes.

Furthermore, is quite striking that a large portion of the healthy controls are classified as showing a decline. The fact that their MoCA scores fall within normal range at baseline argues against the possibility that these healthy controls were actually not healthy. However, since we advertised for participation by mentioning that the study included a thorough evaluation of cognitive performance, we cannot rule out that participants with cognitive concerns participated. This population may therefore resemble the population of patients with subjective cognitive complaints. Neuropsychological differentiation of patients with subjective cognitive complaints from healthy controls requires very sensitive methods to detect subthreshold differences [128].

Finally, it is possible that the automated segmentation is a weakness that should be overcome technically [129]. Single tools differ largely between each other in terms of hippocampal volume. It is possible that this variability contributes to varying extent of usefulness of hippocampal volumetry in the prediction of memory decline [130].

### 4.4. Conclusions

We argue that the prediction of cognitive decline on specific subscales as well as the course of depression is best by the combination of neuropsychological examination, imaging, and neurophysiological assessment and thus by examination techniques that are readily available but not routinely used in clinical practice. The assessment of shared characteristics in neurological populations may open up new perspectives for the development of intelligent diagnostic systems that can be integrated into clinical practice.

The present study tried to provide further evidence for the unifying hypothesis of cognitive decline in temporal lobe epilepsy and dementia or its prodromal stages of subjective cognitive complaints and mild cognitive impairment [23, 131]. However, beca use of the limited sample size, the results can be at most regarded as encouraging for future research.

## Figures and Tables

**Table 1:** Sample characteristics of the subgroups at inclusion time.

Sample	MCI	SCC	TLEr	TLEl	HC
*N*	19	4	6	3	18
Mean age	65.06	67.17	31.85	48.54	57.05
Median age	65	69	28	53	61
Age SD	8.36	7.74	10.54	8.97	14.80
Age range	49–76	56–75	21–50	38–54	24–74
*N* women	8	3	2	3	12
*N* right-handed	19	4	5	3	18

*N* = number; MCI = mild cognitive impairment; SCC = subjective cognitive complaints; TLEr = right-lateralized temporal lobe epilepsy; TLEl = left-lateralized TLE; HC = healthy controls; SD = standard deviation.

**Table 2:** Effects of group, cognitive decline, and interaction between group and decline.

	Group		Decline		Interaction	
	WTS	*p*	WTS	*p*	WTS	*p*
*Wechsler's intelligence test, IQ values*						
Matrices	32.91	<0.001	0.01	0.91	1.27	0.87
Mosaics	33.61	<0.001	1.39	0.24	8.27	0.08
Repeating numbers	25.22	<0.001	0.21	0.65	3.92	0.42
*Regensburg verbal fluency test, RWT, T-values*						
Verbal fluency	70.62	<0.001	0.04	0.85	4.02	0.4
Categorical fluency	48.8	<0.001	11.12	0.001	6.30	0.18
Semantic fluency	85.19	<0.001	2.99	0.08	5.67	0.23
Category transition	273.01	<0.001	0.48	0.49	15.19	0.004
*Verbal memory test, VLMT, T-values*						
Learning	16.15	0.003	2.25	0.13	1.86	0.76
Consolidation	32.2	<0.001	0.55	0.46	1.37	0.85
Recall	15.87	0.003	0.23	0.63	7.28	0.12
Recognition	13.31	0.01	0.08	0.77	5.58	0.23
*Attentional performance, TAP, T-values*						
Flexibility (sum)	5.47	0.24	0.31	0.58	13.31	0.01
Acoustic reaction 1	1.48	0.83	0.36	0.55	2.25	0.09
Visual reaction 1	24.47	<0.001	0.001	0.98	0.49	0.98
Errors 1	7.55	0.11	5.58	0.02	7.07	0.13
Misses 1	7.58	0.11	1.8	0.18	2.89	0.58
Acoustic reaction 2	3.59	0.46	0.006	0.94	5.77	0.22
Visual reaction 2	16.96	0.002	3.28	0.07	3.17	0.53
Errors 2	3.02	0.55	4.13	0.04	4.93	0.30
Misses 2	9.34	0.05	0.99	0.32	2.51	0.64
MWT IQ	12.66	0.005	4.64	0.031	0.36	0.95
DCS, percentile rank	61.88	<0.001	4.60	0.03	13.16	0.01
BDI, sum score	21.15	<0.001	0.34	0.56	1.44	0.84

WTS = Wald-type statistics; MWT = multiple-choice lexical test; DCS = test for cerebral damage; BDI = Beck's Depression Inventory.

**Table 3:** Number of participants per group and per cognitive domain with a decline in cognitive performance of at least one standard deviation on at least one of the included subscales.

Sample	Executive functions	Visual-verbal memory	Divided attention	Depression
Total	50	50	47	47
No change	23	27	24	40
Change	27	23	23	7
MCI				
No change	7	9	8	15
Change	12	10	11	3
SCC				
No change	0	2	2	3
Change	4	2	2	1
TLE right				
No change	4	3	1	4
Change	2	3	2	0
TLE left				
No change	3	3	2	1
Change	0	0	1	2
HC				
No change	9	10	11	17
Change	9	8	7	1

MCI = mild cognitive impairment; SCC = subjective cognitive complaints; TLE = temporal lobe epilepsy; HC = healthy controls.

**Table 4:** Classification results with both sensitivity for decline as well as specificity for no decline >70.

Prediction	Acc	Spec	Sens	MRI	EEG	EEGfeat	PSY
Executive	76	77	75	Structural	Recognition 2	pCOH	Yes
Functions	77	72	82	Structural	Recall 2	pCOH	No
Visual-verbal	80	77	83	Wavelet	No	No	No
Memory	86	95	74	Wavelet	No	No	Yes
Divided	81	84	76	Wavelet	No	No	Yes
Attention	79	79	79	Structural	Rest 2	iCOH	No

Acc = accuracy; Spec = specificity; Sens = sensitivity; MRI = magnetic resonance imaging; EEG = electroencephalography; feat = features; MCI = mild cognitive impairment; SCC = subjective cognitive complaints; TLEr = right-lateralized temporal lobe epilepsy; TLEl = left-lateralized TLE; HC = healthy controls; pCOH = partial coherence; iCOH = imaginary coherence; PDCF = partial directed coherence factor.

## Data Availability

The raw data (EEG, MRI, and neuropsychology) used to support the findings of this study are available from the corresponding author upon request.

## Supplementary Materials

Supplementary Materials This section includes supplementary information for materials and methods, supplementary figures, supplementary tables, and regions of the automated segmentation based on Hammer's atlas.
