# Supplementary material for: Prediction of Cognitive Decline in Temporal Lobe Epilepsy and Mild Cognitive Impairment by EEG, MRI, and Neuropsychology
Source: Comput Intell Neurosci. 2020 May 20;2020:8915961. doi: 10.1155/2020/8915961 (PMC7256687; doi:10.1155/2020/8915961)
Supplement: Supplementary Materials — This section includes supplementary information for materials and methods, supplementary figures, supplementary tables, and regions of the automated segmentation based on Hammer's atlas. [file 8915961.f1.pdf]

Supplementary Material: Prediction of cognitive decline in temporal lobe epilepsy and mild cognitive impairment by EEG, MRI, and neuropsychology

Yvonne Höller[a,b,*], Kevin HG Butz[b], Aljoscha C Thomschewski[b,c], Elisabeth V Schmid[b,c], Christoph D Hofer[e], Andreas Uhl[e], Arne C Bathke[f], Wolfgang Staffen[b], Raffaele Nardone[b,c,d], Fabian Schwimmbeck[b], Markus Leitinger[b], Giorgi Kuchukhidze[b], Marlene Derner[g], Jürgen Fell[g], Eugen Trinka[b]

[a] *Faculty of Psychology, University of Akureyri, Akureyri, Iceland*
[b] *Department of Neurology, Centre for Cognitive Neuroscience, Paracelsus Medical University, Salzburg, Austria*
[c] *Spinal Cord Injury and Tissue Regeneration Center, Paracelsus Medical University, Salzburg, Austria*
[d] *Department of Neurology, F. Tappeiner Hospital, Merano, Italy*
[e] *Multimedia Signal Processing and Security Lab, Department of Computer Sciences, Paris Lodron University, Salzburg, Austria*
[f] *Research Group Statistics and Probability, Department of Mathematics, Paris Lodron University, Salzburg, Austria*
[g] *Department of Epileptology, University of Bonn, Bonn, Germany*

1. Supplementary Informations for Materials and Methods

1.1. Feature vectors for classifications

- Each of the 14 measures of interaction calculated for EEG segments during the two sessions of rest (14×2 classifications).
- Each of the 14 measures during learning, immediate recall, delayed recall after two weeks, immediate recognition, and delayed recognition after two weeks (14×5 classifications).
- Each of the 3 MRI feature vectors (3 classifications).
- Neuropsychological test results at baseline (1 classification).

Then, we created combinations of all of these feature vectors:

- All EEG measures during rest with all MRI feature vectors ($14 \times 2 \times 3$ classifications).
- All EEG measures during cognitive tasks with all MRI feature vectors ($14 \times 5 \times 3$ classifications).
- All EEG measures during rest with the neuropsychological feature vector ($14 \times 2 \times 1$ classifications).
- All EEG measures during cognitive tasks with the neuropsychological feature vector ($14 \times 5 \times 1$ classifications).
- All MRI feature vectors with the neuropsychological feature vector (3×1 classifications).
- All EEG measures during rest with all MRI feature vectors and the neuropsychological feature vector ($14 \times 2 \times 3 \times 1$ classifications).
- All EEG measures during cognitive tasks with all MRI feature vectors and the neuropsychological feature vector ($14 \times 5 \times 3 \times 1$ classifications).

1.2. Feature subset selection

Because of the high dimensionality of the data, we implemented a feature subset selection procedure. Specifically, it is known that when this length exceeds the size of the sample, it can cause artificially high accuracies due to overfitting. This is easily the case for the EEG measures of interaction, because here the length of the feature vector is up to $17 \times 17 \times 6$ for the 17 selected channels and the 6 frequency bands.

Classification and feature subset selection was done in a nested design with 3 layers with 5-fold cross validation (an illustration can be found in Figure 1 in the Supplementary section). We implemented an outer layer as a division of the data into 20% of the data for testing the resulting model, and 80% for feature vector optimisation and cross validation, i.e. submitted to the middle layer. The middle layer is a first inner loop, implemented again with 5-fold cross-validation. This loop aims to estimate the consistency of

*Corresponding author, Faculty of Psychology, University of Akureyri, Nordurslód 2, 600 Akureyri, Iceland
URL: yvonne@unak.is (Yvonne Höller)

selected features, since each run yields a different feature vector. The inner layer is a second, thus, nested inner loop, again with 5-fold cross-validation in order to perform adequate feature subset selection. So-called k-fold cross-validation consist of k repetitions of leaving out N/k samples as the training set, while the remaining $N-(N/k)$ samples are used during the training step.

All subsets were drawn in order to maintain the original proportion of the two groups of participants with vs. without cognitive decline on the respective subscale.

The whole algorithm is described as follows:

1. First, one fifth of the segments were excluded as the outer-layer test set for the final validation step in the outer layer, while the remaining four fifths of segments were used as the outer-layer training set and, thus, submitted to the next step.
2. The outer-layer training set obtained from the outer loop was again divided into 5 equal sized subsets, each one maintaining the proportion of group sizes (with/without decline) from the original sample. For each of these 5 sets, the following steps were repeated:
   (a) The set was left out, the other 4 sets were merged to form the middle-layer training set.
   (b) A t-test for the middle-layer training-set segments was calculated between the two conditions, thus yielding one p-value for each entry of the feature vector.
   (c) The resulting p-values were sorted in ascending order.
   (d) The feature vector was initiated by taking the feature with the smallest p-value, thus, the initial length was one.
   (e) For this feature vector, the classification accuracy was calculated with 5-fold cross-validation, thus, the middle-layer training set was divided into an inner-layer 5-fold partition with an inner-layer training- and testing set
   (f) Now, the next feature from the sorted list was added. For this feature vector, the inner-layer classification with 5-fold cross-validation was repeated.
   (g) Now the result was compared to the previous result. The new entry to the feature vector was included only if the condition constraints were met as follows:
      - The classification accuracy obtained with the current feature vector was $\geq$ the maximum of the previously obtained classification accuracies; that is, the second accuracy had to be $\geq$ than the first entry; for the 6th entry accuracy was compared to the accuracy of the previously obtained feature vector of 5 entries, which is the vector with the maximal accuracy.
      - If the so far best sensitivity/specificity, or in other words, accuracy for segments of the first condition/second condition, respectively, was lower than 0.75, then the obtained sensitivity had to be $\geq$ than this maximum.
      - If the so far best specificity/sensitivity, was lower than 0.5, then the obtained specificity had to be larger, that is $>$ than this maximum.
   (h) This way, features were added and tested for their contribution to the classification accuracy until all available features were used, or until the feature vector reached a maximum of 30 entries, or if more than a consecutive number of 10% of all available features was not added to the feature vector. If 10% was less than 100 features, than the maximum number of features that were tested was 100 or, if the maximum number of available features was lower than 100, the maximum number.
3. The average length N of the resulting 5 optimised feature sets was calculated. The number of times each feature was selected across these 5 runs was counted. A final feature vector was formed by including only those features which were selected at least in 2 of the 5 iterations. If this resulted in no features, all features were included that were selected at least in 1 out of 5 iterations. If the resulting feature vector included more than N features, only the top-most selected 30 features were included. If all features were selected the same amount of times (e.g. one time) a random selection was chosen.
4. The resulting feature vector was used to train a support vector machine on the outer-layer training set, and the resulting model was used to classify the outer-layer test set, which was then used to calculate the general classification accuracy and the within-group accuracy for the two conditions (i.e. sensitivity/specificity).

The threshold of 0.75 was selected as rough estimators for above-chance classification; a value of 0.75 can be considered to be clearly above chance, since the expected chance level would be around 0.5.

1.3. Task

The learning session contained the presentation of 72 pairs of german nouns. The order of the words was kept constant over all participants. Of these pairs of words, 36 had an obvious semantic relationship (such as water - glas), and 36 had no obvious relationship (such as heaven - bookshelf). This variation should ease the remembering for half of the words, while making it more difficult for the second half. First, after presentation of each pair of words, the participant had to indicate whether there was a relationship between the two words or not, by pressing a button on the keyboard. After the button was pressed the participant was prompted on the screen with the question *'Relation between words?'* and in a second line below the instruction *'Please spell out the relationship and press button to continue.'* In this time window, the participant was requested to spell out the potential relationship that came to his or her

mind. This step allowed us to control for the learning strategy employed by the participants. Thinking of a possible relationship should facilitate learning.

The recall session consisted of 72 trials, repeating the 72 word-pairs from the learning phase in the same order. Each trial was formed by a cued recall and a recognition phase. In the cued recall, only the first word was given on the screen, and a question mark indicated that the second word should be reported. Participants proceeded with a button press to the next screen on which they were asked to spell out the second word or to indicate that they had forgotten it. An experimenter took a note on the correctness of the word. Only identical words were considered as correct, with one exception where the plural of a word was accepted as correct (story - stories). After that, a further button press brought the participant to the recognition phase. Here, next to the cue word, three words were presented. The correct word appeared in a pseudo-randomized order on the three positions, and the participants had to select the correct word via button press.

2. Supplementary Figures

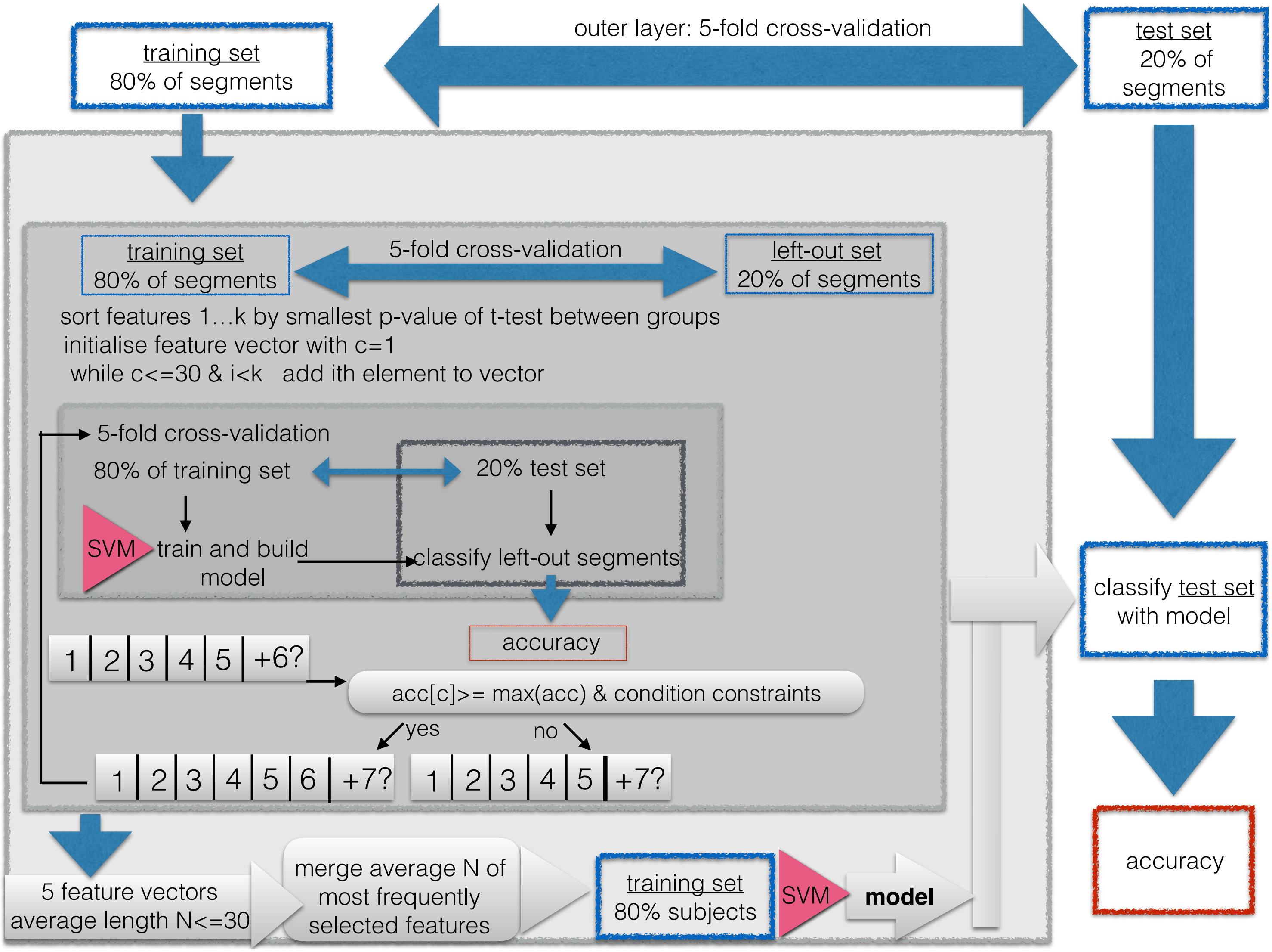

Figure 1: **S1: Classification and feature subset selection procedure.** A nested-cross-validation procedure with an outer-loop for estimation of generalisation and an inner-loop for feature vector optimisation was implemented.

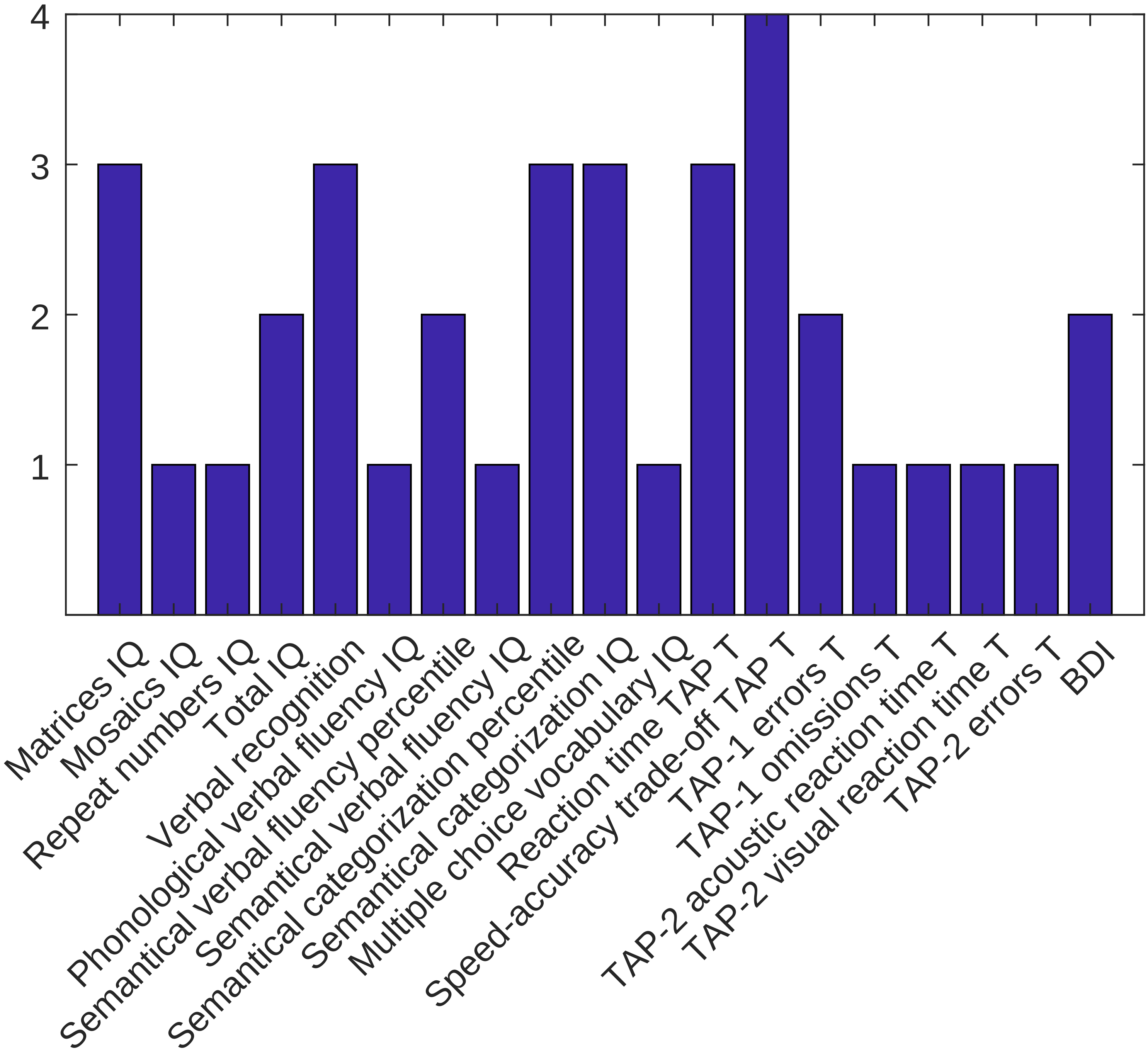

Figure 2: **S2: Neuropsychological scales selected for prediction of executive functions decline.** The bars indicate how often during the cross-validation process a neuropsychological scale was included into the prediction of decline of executive functions. IQ: intelligence quotient; TAP: test for attentional performance; T: T-value; BDI: Beck depression inventory;

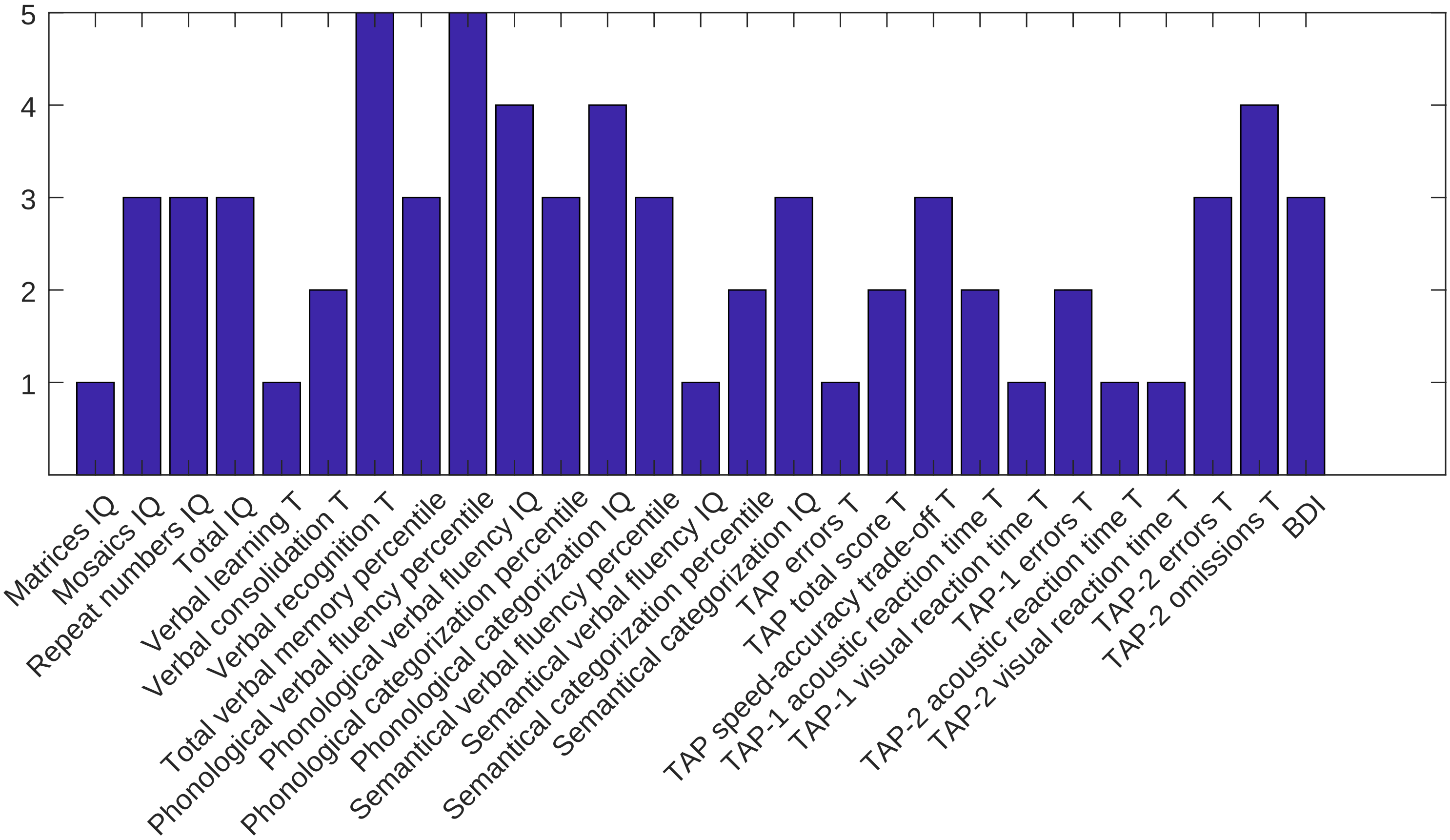

Figure 3: **S3: Neuropsychological scales selected for prediction of increase in depressive symptoms.** The bars indicate how often during the cross-validation process a neuropsychological scale was included into the prediction of decline of executive functions. IQ: intelligence quotient; TAP: test for attentional performance; T: T-value; BDI: Beck depression inventory;

3. Supplementary tables

Table 1: **S1: Demographic data and clinical findings on the hippocampus from structural MRI at baseline**

code	group	age	hand	sex	MRI
MCI01	MCI	74	r	f	left: mild hippocampal atrophy
MCI02	MCI	73	r	m	bilateral hippocampal atrophy
MCI03	MCI	71	r	f	bilateral mild/moderate hippocampal atrophy
MCI04	MCI	73	r	m	normal
MCI05	MCI	76	r	m	bilateral moderate atrophy, left>right
MCI06	MCI	73	r	m	bilateral severe atrophy
MCI07	MCI	61	r	m	bilateral moderate atrophy, left>right
MCI08	MCI	64	r	m	normal
MCI09	MCI	72	r	f	normal
MCI10	MCI	49	r	m	normal
MCI11	MCI	62	r	m	left: hippocampal malrotation
MCI12	MCI	60	r	f	normal
MCI13	MCI	64	r	m	normal
MCI15	MCI	66	r	m	normal
MCI16	MCI	63	r	f	normal
MCI17	MCI	51	r	f	left: mild atrophy
MCI19	MCI	51	r	m	normal
MCI20	MCI	72	r	m	n.a.
MCI21	MCI	69	r	f	normal
MCI22	MCI	57	r/l	f	n.a.
SCC01	SCC	56	r	f	left: moderate hippocampal atrophy
SCC02	SCC	69	r	m	normal
SCC03	SCC	68	r	f	mild bilateral hippocampal atrophy, right>left
SCC05	SCC	75	r	m	bilateral minor hippocampal atrophy, right>left
TLE201	TLEr	50	r	m	right: hippocampal sclerosis
TLE202	TLEr	21	l	m	left: mild hippocampal sclerosis
TLE205	TLEr	37	r	f	left: mild hippocampal sclerosis
TLE207	TLEl	54	r	f	left: hippocampal sclerosis
TLE210	TLEr	29	r	f	right: hippocampal sclerosis
TLE212	TLEl	38	r	f	normal
TLE214	TLEl	53	r	f	left: hippocampal cortical dysplasia, hippocampal sclerosis
TLE216	TLEr	28	r	m	oligodendroglioma grade II, right mesial
TLE217	TLEr	26	r	m	normal
HC01	HC	41	r	m	normal
HC02	HC	67	r	f	bilateral mild hippocampal atrophy
HC04	HC	66	r	m	bilateral mild hippocampal atrophy, left>right
HC05	HC	61	r	m	bilateral mild hippocampal atrophy
HC06	HC	49	r	m	normal
HC07	HC	52	r	f	normal
HC08	HC	66	r	f	left: hippocampal malrotation
HC10	HC	70	r	w	normal
HC13	HC	74	r	m	normal
HC16	HC	67	r	f	normal
HC17	HC	45	r	f	normal
HC18	HC	62	r	f	normal
HC19	HC	26	r	m	normal
HC20	HC	24	r	f	normal
HC21	HC	72	r/l	f	bilateral mild hippocampal atrophy
HC23	HC	61	r	f	bilateral hippocampal atrophy, severe cortical atrophy

Continued on next page

nr	group	age	hand	sex	MRI
HC24	HC	59	r	f	left: mild hippocampal malrotation
HC26	HC	65	r	f	normal

MCI=mild cognitive impairment; SCC=subjective cognitive complaints; TLEr=right-lateralised temporal lobe epilepsy; TLEl=left lateralised temporal lobe epilepsy; HC=healthy controls hand=handedness; r=right; l=left; m=male; f=female; n.a. = information not available;

Table 2: **S2: Self-reported medications of participants at baseline.**

code	group	general	anti-epileptic drugs	psycho-pharmacological drugs
MCI01	MCI	Simvastatin 40mg 1; Enalapril-maleat/Hydrochlorothiazid 1; Vitamin D 2xweek	0	0
MCI02	MCI	Ginko 80mg 1-0-1	0	0
MCI03	MCI	Atenolol/Nifedipin 1-0-1	0	0
MCI04	MCI	Bezastad 200mg 1-0-0, Doxazosin 4mg 1/2-1/2-1/2, Rilmenidine 1mg 1-0-0, Amlodipin 5mg 1-0-1, Nebivolol 5mg 1-0-0, Candesartan Cilexetil/Hydrochlorothiazid 16/12.5mg 1-0-0	0	0
MCI05	MCI	Bisoprolol 1/2-0-1/2; Metformin 850mg; Simvastatin 80mg 1/2; Tamsulosin 0.4mg; Phenoprocoumon, Furadantin 1-0-1	0	0
MCI06	MCI	0	0	0
MCI07	MCI	Simvastatin 20mg every 2 days	0	0
MCI08	MCI	0	0	0
MCI09	MCI	Acenocoumarol 3/4, Sotalol 1, Olmesartanmedoxomil/Hydrochlorothiazid 1, Doxazosin 1	0	0
MCI10	MCI	Lisinopril 20/25mg	0	0
MCI11	MCI	Acetylsalicylic Acid 1, Enalapril-maleat/Hydrochlorothiazid 1x, Metformin 1x	0	0
MCI12	MCI	Ibandronate	0	0
MCI13	MCI	Tiotropium 1x, Beclometasone/Formoterol 2x, Acetylsalicylic Acid 1x, Amlodipin 1x	0	0
MCI15	MCI	Bisoprolol 2.5mg 1-0-0	0	0
MCI16	MCI	Bisoprolol 2.5mg 1-0-1	0	0
MCI17	MCI	Tizanidin 4 mg 1x evening, Diclofenac 50mg rapid, Ginko 80mg 1-0-1	0	0
MCI19	MCI	0	0	0
MCI20	MCI	Valsartan 1x, Valsartan/Hydrochlorothiazid 1x	0	0
MCI21	MCI	Lisinopril/Hydrochlorothiazid 1x, Atorvastatin 1x, Ginko 2x	0	0
MCI22	MCI	0	0	0
SCC01	SCC	Levothyroxin 100mg 1/2-0-3/4; folic acid, b-vitamins	0	Johanicum
SCC02	SCC	Lisinopril/Hydrochlorothiazid 1x	0	0
SCC03	SCC	Valsartan 1x	0	Citalopram 1x, Ginko 2x

Continued on next page

code	group	general	anti-epileptic drugs	psycho-pharmacological drugs
SCC05	SCC	Carvediol 25mg 0-0-1, Gliclazide modified release 30mg 2-0-0, Lisinopril Int 20mg 1-0-0, Lisinopril Hct 25mg 1-0-0, Metformin Rtp 850mg 1-1-1, Simvastatin 30mg 0-0-1, Enalapril/Lercanidipin 10/20mg 0-0-1	0	0
TLE201	TLEr	0	Levetiracetam 2x	0
TLE202	TLEr	0	Lacosamid 200mg 1-1, Lamotrigin 100mg 1-1, Lamotrigin 50mg 0-1	0
TLE205	TLEr	Ibumetin forte 400mg when necessary	Levetiracetam 1000mg 1-0-1, Lacosamid 100mg 1-0-1	0
TLE207	TLEl	Acetylsalicylic acid 100mg	Levetiracetam 3000mg, Lamotrigin 174mg	Trazodon 100mg
TLE210	TLEr	Folic acid 1-0-0	Levetiracetam 1000mg 1-0-1, Levetiracetam 500mg 0-0-1, Lamotrigin 200mg 1-0-1, Lamotrigin 100mg 1-0-1	0
TLE212	TLEl	0	Levetiracetam 1000mg 1-1,	Piracetam 600mg 1 1/2- 1 1/2
TLE214	TLEl	Mexalen 500mg 1-1-1	Levetiracetam 500mg 2-0-2; Perampanel 2mg 0-0-1;	Triazolam 0,25mg 0-0-0-1
TLE216	TLEr	0	Zonisamid 150mg	Cannabis
TLE217	TLEr	?	?	?
HC01	HC	Loratadin 10mg 0-0-1	0	0
HC02	HC	Omeprazol 20mg, Acenocumarol 1/2, Nebivolol 1, Ramipril 1, Bezafibrat, Levothyroxin-Natrium 1	0	0
HC04	HC	Losartan, Losartan HCT, Torasemid, Levothyroxin/Iod, Acetylsalicylic acid	0	0
HC05	HC	0	0	0
HC06	HC	0	0	0
HC07	HC	Thyroxin	0	0
HC08	HC	Dorzolamid/Timolol. 1-0-1, Mefenamin acid when necessary 0-4	0	0
HC10	HC	?	?	?
HC13	HC	Acetylsalicylic acid 1/2,	0	0
HC16	HC	0	0	0
HC17	HC	0	0	0
HC18	HC	Levothyroxin 100mg 1-0-0, Nebivolol 1/2-0-0, Enalaprilmaleat/Lercanidipinhydrochlorid 0-0-1	0	0
HC19	HC	0	0	0
HC20	HC	Levothyroxin 75mg	0	0
HC21	HC	0	0	0
HC23	HC	Lisinopril 2x 1/2, Simvastatin 0-0-1	0	0
HC24	HC	Thyroxin 50mg	0	0

Continued on next page

code	group	general	anti-epileptic drugs	psycho-pharmacological drugs
HC26	HC	Metformin 500mg 2-0-2, Thyroxin 100mg 1/2-0-0, Vildagliptin 50mg 1-0-1	0	0

MCI= mild cognitive impairment; SCC=subjective cognitive complaints; TLEr=right-lateralised temporal lobe epilepsy; TLEl=left lateralised temporal lobe epilepsy; HC=healthy controls

Table 3: **S3: Clinical evaluation of the EEGs of all participants included in this study.**

code	group	EEG1			EEG2		
		**awake**	**base**	**clinical**	**awake**	**base**	**clinical**
MCI01	MCI	yes	10	no	yes	10	no
MCI02	MCI	yes	10	no	yes	10	no
MCI03	MCI	yes	13	no	yes	13	no
MCI04	MCI	yes	11	no	yes	11	no
MCI05	MCI	yes	10	no	yes	10	FS δ T8
MCI06	MCI	yes	10	no	yes	10	FS θ F4
MCI07	MCI	wake N1; vertexwaves; α-dropout	13	no	wake N1; vertexwaves; α-dropout	13	no
MCI08	MCI	wake-N1	9	no	yes	9	no
MCI09	MCI	wake-N1; α-dropout	10	FS θ F7	yes	10	FS θ F7
MCI10	MCI	yes	10	FS δ P8, P7	yes	10	FS δ P8, P7
MCI11	MCI	yes	10	FS δ F7-T7, T8	yes	10	FS δ F7-T7, T8
MCI12	MCI	yes	10	no	yes	10	no
MCI13	MCI	yes	11	no	yes	11	no
MCI15	MCI	yes; α-dropout	9	no	yes; α-dropout	9	no
MCI16	MCI	yes	10	FS δ T7, T8	yes	10	FS δ T7, T8
MCI17	MCI	yes	10	FS θ F7, F8	yes	10	FS θ F7, F8
MCI19	MCI	yes; α-dropout	9	FIRDA	yes; α-dropout	9	FIRDA
MCI20	MCI	yes	10	no	yes	10	no
MCI21	MCI	yes; α-dropout	11	FS δ-θ F7-T7, F8	yes; α-dropout	11	FS δ-θ F7-T7, F8
MCI22	MCI	yes	12	FS δ F7, F8	yes	12	FS δ F7, F8
SCC01	SCC	yes	11	-	yes	11	-
SCC02	SCC	yes	10	-	yes	10	-
SCC03	SCC	yes	13	-	yes	13	-
SCC05	SCC	yes	13	FS θ F8	yes	13	FS θ F8
TLE201	TLEr	yes	10	no	yes	10	no
TLE202	TLEr	yes	10	FS θ F4-F8	yes	10	FS θ F4-F8
TLE205	TLEr	yes	10	repetitive sharp-waves F8-T8: 1.5-2/s	yes	10	no
TLE207	TLEl	yes	10	no	yes	10	no
TLE210	TLEr	yes	10	FS δ F8	yes	10	FS δ F8
TLE212	TLEl	yes	9	no	yes	9	no
TLE214	TLEl	yes	10	no	-	-	-
TLE216	TLEr	yes	12	no	wake-N1	12	no
TLE217	TLEl	yes	12	FS θ F8	-	-	-
HC01	HC	yes	10	no	yes	10	no
HC02	HC	yes	10	no	yes	10	no
HC04	HC	yes	10	no	yes	10	no
HC05	HC	yes	10	no	yes	10	no
HC06	HC	yes	9	no	yes	9	no
HC07	HC	wake-N1	13	no	yes	13	no
HC08	HC	wake-N1	9	no		9	no
HC10	HC	yes	12	no	yes	12	no
HC13	HC	yes	10	no	yes	10	no

Continued on next page

code	group	EEG1			EEG2		
		**awake**	**base**	**clinical**	**awake**	**base**	**clinical**
HC16	HC	yes	13	no	yes	13	no
HC17	HC	yes	10	no	yes	10	no
HC18	HC	wake N1; vertexwaves; α-dropout	12	FS δ-θ T7, T8	yes	12	FS δ-θ T7, T8
HC19	HC	yes	10	no	wake N1; vertexwaves; α-dropout	10	no
HC20	HC	yes	10	no	yes	10	no
HC21	HC	yes	11	FS θ T8	yes	11	FS θ T8
HC23	HC	yes	10	no	yes	10	no
HC24	HC	yes	10	no	yes	10	no
HC26	HC	yes	11	FS δ-θ T7	yes	11	FS δ-θ T7

EEG1/2= results from clinical evaluation of the first and second EEG recording; MCI=mild cognitive impairment; SCC=subjective cognitive complaints; TLEr=right lateralised temporal lobe epilepsy; TLEl=left lateralised temporal lobe epilepsy; HC=healthy controls; awake = wakefulness/ sleep signs or stage; FS = focal slowing; FIRDA = frontal intermitted rhythmic delta activity

Table 4: **S4: Details about the patients with temporal lobe epilepsy included in this study.**

code	lateralisation	localisation	type	seizure
TLE201	right	mesial	focal S/C	no
TLE202	right	mesial	focal S/C, FTSG	n.a.
TLE205	right	mesial	focal C, FTSG	n.a.
TLE207	left	mesial	focal S	yes
TLE210	right	n.d.	focal S	no
TLE212	left	mesial	focal C, FTSG	no
TLE214	left	n.d.	focal C	no
TLE216	right	mesial	focal C, FTSG	no
TLE217	right	mesial/insular	focal S/C	n.a.

TLE= temporal lobe epilepsy; type=seizure type;
seizure=seizures within 24h before/after EEG;
n.a. = information not available; n.d.= not defined
S = simple (without loss of consciousness);
C= complex (with loss of consciousness)
FTSG= focally triggered secondary generalised tonic-clonic seizure

Table 5: S5: Neuropsychological test results of the sub-groups at inclusion time.

	MCI	SCC	TLEr	TLEl	HC
**Wechsler's intelligence test, IQ values**					
Matrices	108	115	96.67	78.33	115.28
Mosaics	101.5	103.75	95	71.67	113.61
repeating numbers	100.6	107.5	88.33	91.67	112.78
**Regensburg verbal fluency test, RWT, T-values**					
verbal fluency	42.68	62.75	18.17	11	63.78
categorical fluency	47.9	74.75	19.83	20	62.72
semantic fluency	63.84	88.75	16	8.33	72.78
category transition	63.58	72	4.83	11	69.89
**verbal memory test, VLMT, T-values**					
learning	47.45	51	47.5	40.33	52.61
consolidation	37.7	40.25	49.33	46.33	45.33
recall	40.55	49.25	42.83	42	50
recognition	41.65	44	43.83	43.67	48.89
**attentional performance, TAP, T-values**					
flexibility (sum)	55.84	51.75	43.75	38.67	55.61
acoustic reaction 1	41.95	44	42.67	37.67	41.39
visual reaction 1	49.47	57.25	47.83	40.33	53.72
errors 1	38	50.25	44.17	35	41.67
misses 1	44.90	42	46	34.33	48.56
acoustic reaction 2	40.78	36	40	29	43.44
visual reaction 2	52	57	32.5	38	52.39
errors 2	40.17	42.25	45.5	37	44.17
misses 2	43.17	43.75	40	33.67	49.33
MWT IQ	103	118	93	107*	116.67
DCS, percentile rank	54.95	55.5	18.83	27	64.83
BDI, sum score	6.89	10.75	15.25	18	3.44

MCI= mild cognitive impairment; SCC= subjective cognitive; complaints; TLEr= right lateralized temporal lobe epilepsy; TLEl= left lateralized TLE; HC= healthy controls; WTS= Wald-type statistics; MWT= Multiple-choice lexical test; DCS= Test for cerebral damage; BDI= Beck's depression inventory

Table 6: **S6: Number of samples for each cognitive subdomain classification result per sub-group of participants without and with decline, after data augmentation.**

domain	MRI	EEG	feature	PSY	no decline	decline
executive	structural	recognition 2	pCOH	yes	357	59
functions	structural	recall 2	pCOH	no	357	59
visual-verbal	wavelet	no	no	no	60	70
memory	wavelet	no	no	yes	243	184
divided	wavelet	no	no	yes	69	49
attention	structural	rest 2	iCOH	no	1346	267
depression	-	rest 1	PDCF	yes	1514	292

PSY= psychological scales included; pCOH= partial coherence;
iCOH= imaginary coherence; PDCF= partial directed coherence factor

Table 7: **S7: Overall accuracy alongside with specificity (percent correctly recognized as showing no decline) and sensitivity (percent correctly recognized as showing decline) separately for neurological populations.**

Prediction	accuracy	MCI		SCC		HC		TLEr		TLEl	
		spec	sens	spec	sens	spec	sens	spec	sens	spec	sens
executive	76	72	62	*	87	63	80	93	100	92	*
functions	77	53	81	*	92	59	77	96	91	90	*
visual-verbal	80	76	100	0	50	100	100	*	*	*	*
memory	86	100	30	0	100	100	100	*	*	*	*
divided	81	50	100	100	100	100	*	*	*	*	*
attention	79	55	86	100	48	90	73	100	85	100	84
depression	83	68	89	76	0	97	96	66	*	100	100

MCI= mild cognitive impairment; SCC= subjective cognitive complaints; TLEr= right lateralised temporal lobe epilepsy;TLEl= left lateralised temporal lobe epilepsy; HC= healthy controls

* no patient available for evaluation after artefact removal

4. Regions of the automated segmentation based on the Hammer's atlas

List of the regions according to the Hammer's atlas. TL: temporal lobe, OL: occipital lobe; CG: cingulate gyrus; FL: frontal lobe; PL: parietal lobe; R: right, L: left;

1. TL hippocampus R
2. TL hippocampus L
3. TL amygdala R
4. TL amygdala L
5. TL anterior temporal lobe medial part R
6. TL anterior temporal lobe medial part L
7. TL anterior temporal lobe lateral part R
8. TL anterior temporal lobe lateral part L
9. TL parahippocampal and ambient gyrus R
10. TL parahippocampal and ambient gyrus L
11. TL superior temporal gyrus middle part R
12. TL superior temporal gyrus middle part L
13. TL middle and inferior temporal gyrus R
14. TL middle and inferior temporal gyrus L
15. TL fusiform gyrus R
16. TL fusiform gyrus L
17. cerebellum R
18. cerebellum L
19. brainstem excluding substantia nigra
20. insula L
21. insula R
22. OL lateral remainder occipital lobe L
23. OL lateral remainder occipital lobe R
24. CG anterior cingulate gyrus L
25. CG anterior cingulate gyrus R
26. CG posterior cingulate gyrus L
27. CG posterior cingulate gyrus R
28. FL middle frontal gyrus L
29. FL middle frontal gyrus R
30. TL posterior temporal lobe L
31. TL posterior temporal lobe R
32. PL inferolateral remainder parietal lobe L
33. PL inferolateral remainder parietal lobe R
34. caudate nucleus L
35. caudate nucleus R
36. nucleus accumbens L
37. nucleus accumbens R
38. putamen L
39. putamen R
40. thalamus L
41. thalamus R
42. pallidum L
43. pallidum R
44. corpus callosum
45. Lateral ventricle excluding temporal horn R
46. Lateral ventricle excluding temporal horn L
47. Lateral ventricle temporal horn R
48. Lateral ventricle temporal horn L
49. Third ventricle

50. FL precentral gyrus L
51. FL precentral gyrus R
52. FL straight gyrus L
53. FL straight gyrus R
54. FL anterior orbital gyrus L
55. FL anterior orbital gyrus R
56. FL inferior frontal gyrus L
57. FL inferior frontal gyrus R
58. FL superior frontal gyrus L
59. FL superior frontal gyrus R
60. PL postcentral gyrus L
61. PL postcentral gyrus R
62. PL superior parietal gyrus L
63. PL superior parietal gyrus R
64. OL lingual gyrus L
65. OL lingual gyrus R
66. OL cuneus L
67. OL cuneus R
68. FL medial orbital gyrus L
69. FL medial orbital gyrus R
70. FL lateral orbital gyrus L
71. FL lateral orbital gyrus R
72. FL posterior orbital gyrus L
73. FL posterior orbital gyrus R
74. substantia nigra L
75. substantia nigra R
76. FL subgenual frontal cortex L
77. FL subgenual frontal cortex R
78. FL subcallosal area L
79. FL subcallosal area R
80. FL pre-subgenual frontal cortex L
81. FL pre-subgenual frontal cortex R
82. TL superior temporal gyrus anterior part L
83. TL superior temporal gyrus anterior part R

5. Funding

The presented research was funded by the Austrian Science Fund (FWF): KLI12-B00 and by the PMU-FFF: A-11/02/004-TRI.
